# Food allergen introduction practices and parent/caregiver attitudes based on family history of food allergy

**DOI:** 10.3389/falgy.2025.1562667

**Published:** 2025-03-19

**Authors:** Hunter G. Smith, Sai Nimmagadda, Ruchi S. Gupta, Christopher M. Warren

**Affiliations:** ^1^ Department of Pediatrics, Lurie Children’s Hospital of Chicago and Northwestern McGaw Pediatrics Residency, Chicago, IL, United States; ^2^ Center for Food Allergy and Asthma Research Northwestern Feinberg School of Medicine & Lurie Children’s Hospital of Chicago, Chicago, IL, United States

**Keywords:** food allergy, food allergy prevention and management, food allergy epidemiology, food allergy prevention guidelines, food allergy family history

## Abstract

**Background:**

The National Institute of Allergy and Infectious Disease (NIAID) addendum guidelines for primary prevention of peanut allergy1 provide recommendations regarding peanut introduction, and a recent consensus statement highlighted the importance of timely introduction of other commonly allergenic solids, and the role of family history as a risk factor.2ObjectiveTo determine whether children in households with a food allergic parent/caregiver or sibling have different rates of being fed commonly allergenic solids during the first year of life than children lacking this family history.

**Methods:**

A pretested survey was administered between January-February 2021 to a U.S. sample of 3,062 parents/caregivers of children born since the NIAID Addendum guidelines. Survey-weighted chi-square statistics and logistic regression models tested the independence of key variables across strata of interest before and after covariate adjustment.

**Results:**

Peanut, almond, shellfish, and other tree nuts are more likely to be introduced to children with one or more food-allergic caregivers. Respondents with food-allergic parents (39.3%) and siblings with FA (35.8%) were more familiar with the 2017 NIAID guidelines compared to parents (12.9%) and siblings without FA (12.7%).

**Conclusion:**

Findings suggest that respondents with food-allergic parents and siblings are more likely to have many of the most prevalent allergens introduced at younger ages, which could be due to knowledge related to the NIAID-sponsored guidelines and other national guidance, but that even among these higher-risk families overall rates of “early” introduction during infancy still remain relatively low.

## 1 Introduction

About 8% of children in the U.S have food allergies with peanuts being the most prevalent allergy affecting an estimated 2.2% of the US pediatric population (1). The other most prevalent allergens include cow's milk (affecting about 1.9%), shellfish (1.3%) (1, 2), tree nuts (1%) (1), and hen's egg (0.8%) (3). As the prevalence of peanut allergy has grown, so have associated rates of healthcare utilization, including for anaphylactic reactions, emergency department (ED) treatment, epinephrine autoinjector prescriptions, and hospitalizations (4, 5). At the same time, management of common food allergies remains suboptimal. For example, even though food allergy-related ED visits are remarkably common among children with shellfish allergy, fewer than half carry an epinephrine autoinjector (2). The LEAP (Learning Early About Peanut Allergy) trial (6) and the National Institute of Allergy and Infectious Disease (NIAID) addendum guidelines for primary prevention of peanut allergy (7), released in 2017, provide evidence-based guidance regarding the optimal timing of peanut introduction. The NIAID-sponsored guidelines suggest that infants with egg allergy or severe eczema be considered for evaluation of peanut-specific IgE through ImmunoCAP testing or skin prick testing to rule out peanut allergy before introducing age-appropriate peanut-containing foods, ideally between 4 and 6 months, in order to reduce the risk of developing peanut allergy. Although these recommendations have been in place for over 5 years, current resident and attending physician knowledge (8) and implementation into clinical practice appears suboptimal (9).

Prior epidemiologic data suggest that children are more likely to develop a food allergy (FA) if they have a parent or sibling with atopic conditions, especially if there is a parental history of food allergies (10, 11). In fact, the role of family history of atopy as an important risk factor for developing FA in infancy was recently reviewed and highlighted in a consensus statement endorsed by the three major North American Allergy Societies summarizing the current state of evidence regarding primary prevention of food allergy via nutritional interventions (11). Despite growing consensus that there exist evidence-based strategies that can prevent specific food allergies in many at-risk infants, FA remains a major public health concern. Even with the accumulating evidence that peanut introduction in the first year of life can prevent peanut allergy, caregivers with and without family history of FA may not be receiving this information from their pediatrician nor be introducing peanuts as recommended. Importantly, a recent pooled analysis of the LEAP and EAT data suggests that regular oral exposure to peanut protein during the first year of life is highly effective for peanut allergy prevention across a variety of risk strata, including among infants with various severities of eczema, levels of peanut-allergic sensitization, and ethnicities (12). Furthermore, many experts now recommend introduction of peanut and egg-containing foods around 6 months (but not before 4 months) and recommended introducing other commonly allergenic foods in that timeframe as well (11).

Whether a child's allergy acquisition is driven primarily by genetics, environmental exposures (e.g., allergen introduction practices), or a combination remains unclear. This effort to characterize the frequency and correlates of parental and sibling FA history, including food allergy-related parental attitudes and behaviors aims to better contextualize current patterns of childhood peanut allergy as well as identify priority populations for implementation of prevention interventions. We hypothesize that children living in households with one or more food allergic parent/caregiver are more likely to be fed peanuts and other commonly allergenic solids during the first year of life than children residing in households where neither parent/caregiver has FA. Our secondary hypothesis is that children living in households with one or more food allergic sibling are more likely to be fed peanuts and other commonly allergenic solids during the first year of life than children residing in households without any food allergic siblings.

## 2 Methods

A multidisciplinary team of pediatricians, pediatric allergists, dieticians, and survey methodologists created a parent/caregiver-report survey to assess current parent/caregiver attitudes and behavior around introduction of specific foods as well as family history of food allergies. Specific foods were identified as the most common food allergens (3) and more popular nuts (e.g., peanuts and almonds) were specifically asked for recall. The survey specifically targeted compliance with the NIAID addendum guidelines. Ethical review and approval was not required for the study on human participants in accordance with the local legislation and institutional requirements as the study was reviewed by the Ann and Robert H. Lurie Children's Hospital of Chicago Institutional Review Board and approved as exempt. After the survey was iteratively refined and reviewed by the multidisciplinary team to ensure all relevant domains were included, a series of cognitive interviews with parents of infants born since the primary prevention of peanut allergy (PPA) guidelines was conducted. The survey was also reviewed by NORC's AmeriSpeak team to ensure high quality instruments were employed. A pronunciation guide and full scripts to train telephone interviewers in English and Spanish was created. The survey was pretested with pilot interviewees and responses were reviewed with NORC. The multidisciplinary team was involved at all phases including finalization of the scripted survey instrument for administration via web and telephone. The survey was subsequently administered in English and Spanish by trained personnel via web and phone between January 21 and February 15, 2021 to a U.S. population-based sample of 3,062 parents/caregivers of children born since the publication of the 2017 NIAID Addendum guidelines (i.e., aged 7–42 months). Written informed consent to participate in this study was provided by the parent/caregiver completing the survey.

### 2.1 Analysis

Subgroup analyses characterized reported introduction of specific allergenic solids by 6, 9, and 12 months of age stratified by caregiver FA status, comparing households with maternal FA, paternal FA, both maternal and paternal FA, or neither maternal nor paternal FA. Similarly, timing of allergenic solid introduction was compared between households where no sibling with FA was present vs. those with one sibling with FA vs. those with multiple siblings with FA. Caregiver knowledge and attitudes were compared using a five-item Likert-scale, comparing caregiver FA and sibling FA subgroups.

Weighted frequencies and proportions were calculated using the svyr package in R 4.1. Survey-weighted chi-square statistics tested the independence of key study variables across strata of interest. All hypothesis testing employed a conventional two-sided *p* < 0.05 level to determine statistical significance. The survey-weighted F statistic was used in the regression analysis to compare variance of samples with the conventional F > 2.5 used to reject the null hypothesis. In the statistical analysis, potential confounders were carefully considered and controlled for in the model as covariates in the analysis. Logistic regression models were fit to assess the degree to which inferences were robust to statistical adjustment for key covariates commonly recognized as potential confounders or significant factors influencing the outcomes of interest (i.e., race/ethnicity, educational attainment, household income, child history of eczema, and if the child was seen by an allergist).

## 3 Results

### 3.1 Sample demographic characteristics

After applying complex survey weights the majority of respondents were between 30 and 44 years of age (58.6%) and 49.3% identified as the child's mother (Table 1). Race and ethnicity were reported in mutually exclusive categories, with 49.4% identifying as non-Hispanic White, 27.9% as Hispanic, and 13.9% as non-Hispanic Black. Of respondents, 28.2% highest educational attainment was high school diploma and 24.5% completed a 2-year (e.g., vocational) degree. Of the child's biological parents, 63.1% of mothers and 62.9% of fathers had no atopic diseases whereas 7.8% of mothers and 5.3% of fathers had food allergies.

**Table 1:** Demographic distribution.

Variable	%
Race and/or ethnicity	
Asian, non-hispanic	4.2%
Black, non-hispanic	13.9%
Hispanic	27.9%
Multi-racial, non-hispanic	3.1%
Other, non-hispanic	1.4%
White, non-hispanic	49.4%
Relationship to respondent	
Mother	49.3%
Father	46.3%
Other	4.4%
Respondent age (in years)	
18–29	33.3%
30–44	59.6%
45–59	5.7%
60+	1.4%
Respondent educational attainment	
Postgraduate/professional	12.9%
Bachelor's degree	19.9%
Vocational tech school	24.5%
High school graduate	28.2%
<High school	14.4%
Atopic disease history of child's biological parents	
Food allergy (mother)	7.8%
Food allergy (father)	5.3%
Eczema (mother)	9.1%
Eczema (father)	5.8%
Asthma (mother)	9.5%
Asthma (father)	9.0%
Environmental allergies (mother)	17.5%
Environmental allergies (father)	18.6%
None (mother)	63.1%
None (father)	62.9%

### 3.2 Patterns of allergenic solid Introduction by parental history of food allergy

Peanut introduction practices differed between families with maternal and/or paternal food allergies. While the differences were not significantly different at 6 months of age, children whose parents had a FA were more likely to introduce peanuts to their children by 9 and 12 months (Table 2). When adjusting for race/ethnicity, educational attainment, household income, child history of eczema, and if the child sees an allergist; this remained significant for peanut introduction by 9 months in children whose fathers had a food allergy. Likewise, almond introduction by 6, 9, and 12 months was more common for children when both maternal and paternal FA was present. For instance, 18.2% of children whose parent did not have FA were introduced to almonds by 12 months compared to 39.8% of children with both maternal and paternal FA (Table 2). This remained significant when adjusting for covariates with introduction of almonds at 12 months for children with both maternal and paternal FA odds ratio (OR) 2.55 [95%CI (1.31–4.94), *p* = 0.01]. The same holds true for any tree nut introduction at the studied ages. Only 25.7% of children whose parent did not have FA were introduced to any tree nut by 12 months as opposed to 47.7% of children with both maternal and paternal FA (Table 2). After adjusting for covariates, tree nut introduction by 12 months remained significantly more likely in households where maternal and paternal FA was present [OR 2.20, 95% CI (1.14–4.25), *p* = 0.02].

**Table 2:** Allergen introduction timing by maternal/paternal FA status.

		Neither maternal nor paternal FA		Maternal FA		Paternal FA		Both maternal and paternal FA		Survey-weighted F statistic	Un-adjusted omnibus *P* Value	Adjusted *P* value for maternal FA	Adjusted *P* value for paternal FA	Adjusted *P* value for maternal + paternal FA
Allergen	Introduced By:	*N*	%	*N*	%	*N*	%	*N*	%					
Peanut	6 months	138	5.1	13	6.4	7	5.5	6	14.3	1.7	0.160	0.56	0.19	0.28
	9 months	757	28.0	69	35.0	45	37.1	16	39.3	2.7	0.045^a^	0.68	0.02	0.08
	12 months	1,176	43.5	101	51.6	69	56.7	25	58.8	3.9	0.009^b^	0.17	0.35	0.58
Milk	6 months	505	18.7	43	22.2	18	14.6	8	18.5	0.9	0.458	0.35	0.20	0.58
	9 months	1,019	37.7	84	43	38	31.4	17	40.6	1.2	0.324	0.30	0.19	0.95
	12 months	1,668	61.7	127	64.8	76	63	26	61.9	0.2	0.889	0.57	0.96	0.73
Shellfish	6 months	56	2.1	6	2.9	1	0.7	3	6.9	2.0	0.122	0.51	0.16	0.25
	9 months	192	7.1	16	8.4	9	7.2	9	20.9	3.1	0.026^a^	0.89	0.91	0.01
	12 months	413	15.3	32	16.4	23	19.3	15	35.3	3.6	0.013^a^	0.80	0.38	<0.001
Almond	6 months	75	2.8	3	1.3	3	2.6	5	11.9	5.5	0.001^a^	0.10	0.72	0.01
	9 months	234	8.7	17	8.6	12	9.8	11	24.5	4.2	0.006^a^	0.73	0.89	<0.001
	12 months	492	18.2	34	17.5	24	19.8	17	39.8	3.2	0.024^a^	0.44	0.97	0.01
Any tree nut	6 months	116	4.3	7	3.8	4	3.5	7	15.9	5.1	0.002^a^	0.63	0.46	0.01
	9 months	333	12.3	30	15.1	16	13.6	12	29.5	3.6	0.014^a^	0.51	0.99	0.01
	12 months	695	25.7	47	23.8	43	35.3	20	47.7	3.9	0.010^a^	0.36	0.18	0.02
Egg	6 months	412	15.2	27	13.8	33	26.8	5	11.8	3.0	0.033^a^	0.56	0.02	0.43
	9 months	1,212	44.8	96	48.9	65	53.7	16	38.6	1.4	0.245	0.59	0.16	0.48
	12 months	1,776	65.7	136	69.6	95	78.7	25	58.8	2.9	0.034^a^	0.33	0.01	0.93

Logistic regression models remained statistically significant after adjusting for key covariates.

^a^ Statistical significance remained in some but not all subgroups after adjustment for key covariates.

^b^ No longer statistically significance after adjusting for key covariates in logistic regression model.

Shellfish introduction was significantly more likely by 9 and 12 months for children with both maternal and paternal FA with an over two-fold increase in introduction by 12 months compared to children whose parents did not have FA. However, when adjusting for the prespecified set of covariates, this difference in rates of shellfish introduction was attenuated below the threshold for statistical significance. Interestingly, eggs were significantly more likely to be introduced by 6 (26.8%) and 12 (78.7%) months for children with paternal caregiver FA compared to children with no caregiver FA by 6 months (15.2%) and 12 months (65.7%). On the other hand, cow's milk introduction was not significantly different among families with and without a parental history of food allergies either with or without covariate adjustment.

### 3.3 Patterns of allergenic solid introduction by sibling history of food allergy

Timing of peanut introduction was not significantly different among children with one or more older sibling with food allergy (Table 3). In contrast, tree nuts were introduced earlier among children with at least one food-allergic sibling. For instance, almond introduction was significantly more common by 6, 9, and 12 months of age for children who had one or multiple siblings with food allergies (Table 3), but this did not remain significant when adjusting for race/ethnicity, educational attainment, household income, child history of eczema, and if the child saw an allergist. Similarly, introduction of any tree nut was significantly higher for 9 month and 12 month olds with one or more siblings with FA but did not remain significant when adjusted for the aforementioned covariates. Shellfish were more likely to be introduced among children with one (23.1%) or multiple siblings (27.1%) with food allergies by 12 months of age compared to no sibling (15.4%) with FA, but again this difference was attenuated below the threshold of statistical significance after covariate adjustment. Milk and egg introduction were not significantly different among children with siblings with FA compared to no siblings with FA in either bivariate or covariate-adjusted analyses.

**Table 3:** Allergen introduction timing by sibling FA status.

		No sibling with FA		One sibling with FA		Multiple siblings with FA		Survey-weighted F statistic	Un-adjusted omnibus *P* Value	Adjusted *P* value for One sibling with FA	Adjusted *P* value for multiple siblings with FA
Allergen	Introduced By:	*N*	%	*N*	%	*N*	%				
Peanut	6 months	409	17.1	22	19.4	6	20.2	0.3	0.750	0.71	0.73
	9 months	1,047	35.8	40	35.8	13	47.2	0.8	0.440	0.19	0.90
	12 months	1,714	58.7	68	60.7	19	64.9	0.3	0.750	0.54	0.97
Milk	6 months	546	18.7	26	23.2	2	8.2	2.3	0.110	0.76	<0.001
	9 months	1,098	37.6	49	44	11	38.6	0.9	0.400	0.42	0.69
	12 months	1,806	61.8	75	67.4	15	53.6	1.1	0.330	0.55	0.27
Shellfish	6 months	62	2.1	3	2.8	1	2.8	0.3	0.700	0.85	0.96
	9 months	211	7.2	11	9.5	3	12.2	1.4	0.240	0.78	0.96
	12 months	449	15.4	26	23.1	8	27.1	4.7	0.010^a^	0.18	0.79
Almond	6 months	79	2.7	6	5.3	1	4.1	2.5	0.09^a^	0.22	0.99
	9 months	252	8.6	16	13.9	6	20.3	5.8	0.003^a^	0.27	0.25
	12 months	530	18.1	27	24.5	10	33.6	4.7	0.009^a^	0.58	0.28
Any tree nut	6 months	124	4.3	8	6.9	2	8.7	2.4	0.90	0.30	0.37
	9 months	364	12.4	20	18.1	7	24.8	4.8	0.008^a^	0.50	0.36
	12 months	756	25.9	37	33.4	11	40.9	3.5	0.030^a^	0.51	0.47
Egg	6 months	453	15.5	21	18.4	2	7.7	1.3	0.260	0.70	0.09
	9 months	1,332	45.6	49	43.6	8	26.6	2.3	0.100	0.46	0.02
	12 months	1,947	66.6	71	63.9	14	50.1	1.8	0.160	0.73	0.26

Logistic regression models remained statistically significant after adjusting for key covariates. Statistical significance remained in some but not all subgroups after adjustment for key covariates.

^a^ No longer statistically significance after adjusting for key covariates in logistic regression model.

### 3.4 Caregiver knowledge and attitudes regarding food allergy prevention by parental FA history

Caregiver-reported attitudes and knowledge regarding introduction of allergenic solids for food allergy prevention differed significantly by parent/caregiver FA status. Among households where both parents/caregivers had FA, 39.3% of respondents were aware of the 2017 NIAID recommendations compared to 12.9% of those where neither parent reported FA (Table 4). Caregivers with FA were significantly more worried about their baby developing FA. Among respondents in households where neither caregiver had FA, 3.7% were “extremely worried” about their baby developing FA compared to 26.7% of respondents in households where both caregivers were food-allergic; a difference which remained highly statistically significant when adjusting for covariates. Likewise, 23.4% of respondents in households where both caregivers were food-allergic “very much” thought that feeding peanut-containing foods in the first year of life would decrease the risk of their child developing peanut allergy. This contrasts with 14.1% of respondents among households where neither caregiver reported FA. When adjusting for covariates, this difference remained statistically significant only for those with paternal FA history [OR 1.92, 95% CI (1.13, 3.25), *p* = 0.02].

**Table 4:** Caregiver knowledge and attitudes by maternal/paternal FA Status.

Survey question	Response options	Neither maternal nor paternal FA		Maternal FA		Paternal FA		Both maternal and paternal FA		Survey-weighted F statistic	Un-adjusted omnibus *P* Value	Adjusted *P* value for maternal FA	Adjusted *P* value for paternal FA	Adjusted *P* Value for maternal + paternal FA
		N	%	N	%	N	%	N	%					
In 2017, an expert panel working with the NIAID recommended that babies with eczema should be fed peanut-containing foods around 4–6 months in order to prevent peanut allergy. Have you heard about this recommendation?	Yes	346	12.9	30	15.4	11	9.4	16	39.3	3.9	0.002^a^	0.87	0.06	0.01
	No	1,902	71	136	70.5	90	74.7	24	57.9					
	Not Sure	431	16.1	27	14	19	15.8	1	2.8					
As you were preparing to begin feeding your baby solid foods, how worried were you about your baby developing a food allergy?	Extremely worried	99	3.7	17	8.6	10	8.4	11	26.7	7.9	<.0001^a^	0.13	0.16	<0.001
	Somewhat worried	266	9.9	23	11.7	14	11.5	8	17.9					
	A little bit worried	870	32.3	73	37.2	40	32.7	8	18.8					
	Not worried at all	1,459	54.2	83	42.5	57	47.4	5	36.6					
Do you think that feeding peanut-containing foods in the first year of life will decrease the risk of your child developing a peanut allergy?	Yes, very much so	381	14.1	40	20.6	29	23.8	10	23.4	2	0.03^a^	0.21	0.02	0.93
	Yes, somewhat	567	21	54	27.7	23	18.8	13	31.3					
	Yes, minimally	300	11.1	19	9.6	14	11.7	7	17.6					
	No, not at all	521	19.3	27	13.8	20	16.7	4	9.6					
	I do not know	926	34.3	55	28.3	35	29	8	18.1					

Logistic regression models remained statistically significant after adjusting for key covariates. No longer statistically significance after adjusting for key covariates in logistic regression model.

^a^ Statistical significance remained in some but not all subgroups after adjustment for key covariates.

### 3.5 Caregiver knowledge and attitudes regarding food allergy prevention by sibling FA history

Caregiver attitudes and knowledge differed significantly among caregivers with other children with FA. For children with multiple siblings with FA, 35.8% of caregivers were aware of the 2017 NIAID recommendations compared to 12.7% of children with no siblings with FA (Table 5); but this difference did not remain statistically significant when adjusting for covariates. For caregivers without other children with FA, 4% were extremely worried about their baby developing FA compared to 25.7% of caregivers with multiple other children with FA. When adjusting for the covariates, having multiple siblings with food allergies remained significantly associated with parental worry about their infant developing FA [OR 2.29, 95% CI (1.11, 4.70), *p* = 0.02]. Likewise, 27.7% of caregivers with multiple other children with FA “very much” thought that feeding peanut-containing foods in the first year of life would decrease the risk of their child developing a peanut allergy compared to 14.6% of caregivers without any other children with FA, but did not remain statistically significant upon covariate adjustment.

**Table 5:** Caregiver knowledge and attitudes by sibling FA Status.

Survey question	Response options	No sibling with FA		One sibling with FA		Multiple siblings with FA		Survey-weighted F statistic	Un-adjusted omnibus *P* Value	Adjusted *P* value for one sibling with FA	Adjusted *P* value for multiple siblings with FA
		*N*	%	*N*	%	*N*	%				
In 2017, an expert panel working with the NIAID recommended that babies with eczema should be fed peanut-containing foods around 4–6 months in order to prevent peanut allergy. Have you heard about this recommendation?	Yes	368	12.7	26	23.4	10	35.8	5.8	0.0002^b^	0.48	0.48
	No	2,065	71.3	73	65.6	15	54.8				
	Not Sure	464	16	12	11	3	9.4				
As you were preparing to begin feeding your baby solid foods, how worried were you about your baby developing a food allergy?	Extremely worried	117	4	12	11.2	7	25.7	10.8	<0.0001^a^	0.47	0.02
	Somewhat worried	289	9.9	15	13.2	6	22.3				
	A little bit worried	943	32.4	40	35.6	7	26.1				
	Not worried at all	1,563	53.7	45	40	7	25.9				
Do you think that feeding peanut-containing foods in the first year of life will decrease the risk of your child developing a peanut allergy?	Yes, very much so	424	14.6	28	25.2	8	27.7	2.4	0.02^b^	0.15	0.90
	Yes, somewhat	622	21.3	27	24.4	8	28.2				
	Yes, minimally	325	11.2	11	10.2	4	12.9				
	No, not at all	549	18.8	21	19.3	4	13.2				
	I do not know	996	34.2	23	21	5	17.9				

Logistic regression models remained statistically significant after adjusting for key covariates.

^a^ Statistical significance remained in some but not all subgroups after adjustment for key covariates.

^b^ No longer statistically significance after adjusting for key covariates in logistic regression model.

### 3.6 Allergenic food introduction timing by parental attitudes and awareness

Parental awareness of PPA guidelines and attitudes toward early allergen introduction were significantly different among parents of children introduced to commonly allergenic solids during their first year. For children introduced to any tree nut by 12 months, 38.1% of their caregivers were aware of PPA guidelines and 39.0% “very much” thought early allergen introduction would prevent food allergy (Table 6), which remained significant after covariate-adjustment. Similarly, for children introduced to peanut by 12 months, 76.3% of their caregivers were aware of PPA guidelines and 84.2% “very much” thought early peanut introduction would prevent peanut allergy, which again retained statistical significance after covariate-adjustment. Children introduced to milk and egg by 12 months had similar trends where over 70% of their caregivers “very much” thought early allergen introduction would prevent food allergy. When adjusting for covariates, the results remained significant with OR 1.91 [95% CI (1.39–2.62), *p* < 0.001]. For those with egg and milk introduced by 12 months 77.3% and 69.2%, respectively, of caregivers “very much” thought early peanut introduction would prevent peanut allergy, both of which remain statistically significant when adjusting for covariates. Milk introduction by 12 months had an OR 1.43 [95% CI (1.08–1.88), *p* = 0.01].

**Table 6:** Allergenic food introduction by parental attitudes and awareness of PPA guidelines.

		Aware of PPA guidelines—answered “yes”				“Very much” think early peanut introduction will prevent peanut allergy				“Very much” think early allergen introduction will prevent food allergy			
Specific Food allergen	Introduced By:	*N*	%	Unadjusted *P* value	Adjusted *P* value	*N*	%	Unadjusted *P* value	Adjusted *P* value	*N*	%	Unadjusted *P* value	Adjusted *P* value
Peanut	6 months	125	31.0	<0.001^a^	<0.001	159	34.6	<0.001^a^	<0.001	145	36.2	<0.001^a^	<0.001
	9 months	242	59.9	<0.001^a^	<0.001	271	58.8	<0.001^a^	<0.001	240	60.0	<0.001^a^	<0.001
	12 months	308	76.3	<0.001^a^	<0.001	388	84.2	<0.001^a^	<0.001	332	83.0	<0.001^a^	<0.001
Milk	6 months	78	19.4	0.78	0.95	99	21.6	0.16	0.16	92	22.9	0.06	0.05
	9 months	171	42.3	0.11	0.23	200	43.4	0.03^b^	0.06	190	47.5	<0.001^a^	<0.001
	12 months	267	66.2	0.12	0.25	319	69.2	0.01^a^	0.01	299	74.7	<0.001^a^	<0.001
Shellfish	6 months	13	3.2	0.26	0.15	15	3.2	0.23	0.06	17	4.7	0.03^a^	<0.001
	9 months	56	13.8	<0.001^b^	0.15	61	13.2	<0.001^a^	<0.001	61	24.1	<0.001^a^	<0.001
	12 months	110	27.2	<0.001^a^	<0.001	118	25.5	<0.001^a^	<0.001	107	26.2	<0.001^a^	<0.001
Almond	6 months	24	5.8	<0.001^a^	0.01	22	4.7	0.03^a^	0.01	20	5.0	0.02^a^	0.01
	9 months	68	16.7	<0.001^a^	<0.001	78	17.0	<0.001^a^	<0.001	61	15.3	<0.001^a^	<0.001
	12 months	129	32.0	<0.001^a^	<0.001	141	30.6	<0.001^a^	<0.001	122	30.4	<0.001^a^	<0.001
Any tree nut	6 months	34	8.4	<0.001^a^	<0.001	33	7.1	0.01^a^	<0.001	30	7.5	0.003^a^	<0.001
	9 months	92	22.8	<0.001^a^	<0.001	92	19.9	<0.001^a^	<0.001	79	19.7	<0.001^a^	<0.001
	12 months	154	38.1	<0.001^a^	<0.001	174	37.8	<0.001^a^	<0.001	156	39.0	<0.001^a^	<0.001
Egg	6 months	78	19.2	0.07^a^	0.03	111	24.1	<0.001^a^	<0.001	98	24.5	<0.001^a^	<0.001
	9 months	213	52.8	0.01^a^	0.01	270	58.6	<0.001^a^	<0.001	154	38.6	<0.001^a^	<0.001
	12 months	281	69.5	0.23	0.22	356	77.3	<0.001^a^	<0.001	316	79.0	<0.001^a^	<0.001

^a^ Logistic regression models remained statistically significant after adjusting for key covariates.

^b^ No longer statistically significance after adjusting for key covariates in logistic regression model.

## 4 Discussion

Based on these nationally representative survey data reported by parents and caregivers of children born since the 2017 publication of the NIAID-sponsored Addendum Guidelines for the Prevention of Peanut Allergy; peanut, almond, shellfish, and other tree nut introduction is more likely for children with one or more food-allergic parents/caregivers. Almond and tree nuts are about twice as likely to be introduced at 6, 9, and 12 months to children with multiple siblings with food allergies compared to those who have no siblings with food allergies. While this suggests that targeted FA prevention messaging may be reaching these households with infants who are at elevated FA risk (11), the observed rates of introduction of these specific allergenic solids are still far below what would be expected with effective national implementation of the PPA guidelines.

Respondents with food-allergic parents and sibling with FA were more likely to report awareness of the 2017 NIAID PPA guidelines compared to parents and siblings without FA. Of the families that reported awareness, three-fourths had introduced peanuts to their child by 12 months; however, it is notable that this PPA guideline-aware group only comprises a small minority (13%) of US households. Even among these higher-risk families, the overall rates of early allergen introduction during infancy remains low. Strikingly, when adjusting for covariates, paternal FA, but not maternal or both paternal and maternal FA, remained statistically significant for thinking that feeding peanut-containing food products in the first year of life would “very much so” decrease the risk of developing peanut allergy. While this brings to question the influence of which caregiver has a FA, past studies have not always shown strong differences between caregiver history, as cited by Saito-Abe, et al., 2022 (13). Other studies have conflicting results on maternal or paternal influence with Koplin et al., 2013 (10) concluding maternal history of allergic disease was more strongly associated with infant food allergy development, but Al-Hammadi et al., 2011 (14) found that paternal allergic rhino-conjunctivitis was also a strong predictor.

Respondents in households with at least one food-allergic parent or sibling also reported higher levels of worry about their child developing a food allergy with over a quarter reporting they were “extremely worried” compared to fewer than 1 in 20 respondents from households with no food allergic caregivers or siblings. Despite their elevated levels of worry, caregivers with FA and other children with FA were significantly more likely to introduce almonds and tree nuts to their child during the first year of life. The availability of various nut-based products, such as almond milk, could play a causal role in this increase. The foods used for introduction were not documented in this study, but provides an area of future analysis and a potential further avenue for early allergen introduction. Although the probability of peanut introduction was not significantly different between households with and without a food-allergic sibling, peanut was introduced significantly more often by 9 and 12 months old in households where at least one or two caregivers reported FA. Shellfish introduction at 12 months also increased significantly for families with food allergic parents. This increase in peanut, shellfish, almond, and overall tree nut introduction could be due to parental increased awareness and motivation to prevent FA in their children—possibly in part due to greater opportunities for clinical food allergy education and PPA guideline dissemination among clinical food allergy care providers or through other food allergy advocacy and educational modalities.

The dramatically elevated levels of worry about developing food allergies reported by food-allergic caregivers or caregivers with other food-allergic children may also serve to increase motivation for food allergen introduction. These subgroups believe that feeding peanut-containing products in the first year of life will decrease the risk of developing a peanut allergy. These data align with other recently published data from this same survey, which revealed less than 1% of caregivers reported feeding their infant an early food allergen introduction product. The infants that were fed these products were more likely to have seen an allergist or have a parent with food allergy (15). Pediatricians and allergists can leverage this information to further encourage caregivers to thoughtfully introduce major allergens within the first 12 months of life. Note that, “early introduction” as recommended by current guidelines does not entail simply feeding a food a single time, but rather involves repeated exposures of protein delivered in developmentally appropriate formats, in sufficient quantities, and at adequate frequencies to induce a tolerogenic immune response. Given the nuances of not only food allergy introduction but also the importance of diet (16) and oropharyngeal development (17) for appropriate pediatric neurodevelopment, especially for preterm infants (18), and microbiome development (19); further cost-benefit evaluation is warranted to determine if healthcare clinics would benefit from increasing access to nutritionist or other dietary support for families of children at increased food allergy risk. Such efforts could emphasize the importance of early allergen introduction and discuss healthy dietary habits, especially healthy alternatives for children with allergies.

Parents reporting awareness of the PPA guidelines were more likely to have introduced peanuts by 12 months. Since caregivers that have been appropriately counseled for peanut introduction are more likely to introduce these foods, more time should be spent in primary care appointments discussing the importance of appropriate introduction of allergenic solids for primary prevention of food allergy. Given the limited time and competing issues commonly addressed during pediatric well-child checks, provider education—including of allied health professionals—and easily obtainable resources could help facilitate effective and efficient counseling. The majority of caregivers that introduce milk and eggs and about one-third that introduce almonds and other tree nuts by 12 months believe early allergen introduction will prevent FA. Primary care providers and allergists should emphasize this information as tree nut (20, 21) and shellfish (21) allergies generally present among infants after 12 months of age.

Although strengths of this study include its large, nationally-representative sample, it is limited by recall bias by parents for PPA guidelines, pediatrician recommendations, and timing of allergen introduction. Specifically, families with other children with FA or parental FA are potentially more likely to remember early allergen introduction practices compared to families without FA. The study is also limited by potential generalizability to other cultures or geographical contexts. Despite several important results being identified, this cross-sectional study is limited in making a causal inference. Although increased familiarity with the NIAID guidelines could plausibly influence earlier allergen introduction; as the data was self-reported, results are also subject to various types of response bias such as social desirability bias.

Given these results, public health efforts can focus on increasing awareness around PPA guidelines and other evidence-based food allergy prevention approaches to limit the increasing healthcare costs and morbidity surrounding FA (4, 5). The efforts can target caregivers with children 4–11 months of age and may include a variety of channels, including patient handouts, waiting room educational materials and multimedia public service announcements. These data indicate that many caregivers with FA and with other children with FA believe feeding peanut-containing foods in the first year of life will decrease the risk of their child from developing a peanut allergy and therefore may be particularly amenable to intervention with appropriate guidance.

Given growing evidence for the effectiveness of early dietary intervention for peanut allergy prevention (22), further work is needed to bring families and food allergy experts into alignment regarding the perceived strength of this evidence, as fewer than 1 in 3 families reported high levels of confidence in the effectiveness of early peanut introduction for peanut allergy prevention. This study provides further evidence of needed early peanut introduction, despite several barriers to implementation since the NIAID guidelines in 2017 (23). While level 1 evidence is admittedly less robust for other foods (e.g., seafood, specific tree nuts, sesame), current consensus approaches recommend timely introduction of those foods as well, given the lack of evidence against its efficacy, growing evidence in support, and demonstrated safety (24). In sum, this study provides epidemiologic findings to improve the public health understanding of family history's influence on childhood FA. Our findings give an insight into the thoughts, feelings, and motivation for food-allergic caregivers and their efforts into preventing FA in their babies. This can further fashion specific discussion topics for motivational counseling for allergists and pediatricians.

## Data Availability

The raw data supporting the conclusions of this article will be made available by the authors, without undue reservation.

## References

[1] Gupta RS Warren CM Smith BM Blumenstock JA Jiang J Davis MM The public health impact of parent-reported childhood food allergies in the United States. Pediatrics. (2018) 142(6):e20181235. 10.1542/peds.2018-1235 30455345 PMC6317772

[2] Wang HT Warren CM Gupta RS Davis CM. Prevalence and characteristics of shellfish allergy in the pediatric population of the United States. J Allergy Clin Immunol Pract. (2020) 8(4):1359–70. 10.1016/j.jaip.2019.12.027 31917365 PMC7951995

[3] Gupta RS Springston EE Warrier MR Smith B Kumar R Pongracic J The prevalence, severity, and distribution of childhood food allergy in the United States. Pediatrics. (2011) 128(1):e9–17. 10.1542/peds.2011-0204 21690110

[4] Warren CM Jiang J Gupta RS. Epidemiology and burden of food allergy. Curr Allergy Asthma Rep. (2020) 20(2):6. 10.1007/s11882-020-0898-7 32067114 PMC7883751

[5] Gupta R Holdford D Bilaver L Dyer A Holl JL Meltzer D. The economic impact of childhood food allergy in the United States. JAMA Pediatr. (2013) 167(11):1026. 10.1001/jamapediatrics.2013.2376 24042236

[6] Du Toit G Roberts G Sayre PH Bahnson HT Radulovic S Santos AF Randomized trial of peanut consumption in infants at risk for peanut allergy. N Engl J Med. (2015) 372(9):803–13. 10.1056/NEJMoa1414850 25705822 PMC4416404

[7] Togias A Cooper SF Acebal ML Assa'ad A Baker JR Beck LA Addendum guidelines for the prevention of peanut allergy in the United States: report of the national institute of allergy and infectious diseases-sponsored expert panel. J Allergy Clin Immunol. (2017) 139(1):29–44. 10.1016/j.jaci.2016.10.010 28065278 PMC5226648

[8] Sandhu S Hanono M Nagarajan S Vastardi MA. Knowledge assessment of early peanut introduction in a New York city population. Ann Allergy Asthma Immunol. (2022) 129(3):380–82. 10.1016/j.anai.2022.06.013 35728745

[9] Warren C Nimmagadda S Samady W Venter C Galic I Hultquist H P104 current US parent/caregiver knowledge, attitudes, and behaviors regarding dietary introduction of peanut protein during infancy. Ann Allergy Asthma Immunol. (2021) 127(5):S40–1. 10.1016/j.anai.2021.08.122

[10] Koplin J Allen K Gurrin L Peters RL Lowe AJ Tang MLK The impact of family history of allergy on risk of food allergy: a population-based study of infants. Int J Environ Res Public Health. (2013) 10(11):5364–77. 10.3390/ijerph10115364 24284354 PMC3863850

[11] Fleischer DM Chan ES Venter C Spergel JM Abrams EM Stukus D A consensus approach to the primary prevention of food allergy through nutrition: guidance from the American academy of allergy, asthma, and immunology; American college of allergy, asthma, and immunology; and the Canadian society for allergy and clinical immunology. J Allergy Clin Immunol Pract. (2021) 9(1):22–43.e4. 10.1016/j.jaip.2020.11.002 33250376

[12] Logan K Bahnson HT Ylescupidez A Beyer K Bellach J Campbell DE Early introduction of peanut reduces peanut allergy across risk groups in pooled and causal inference analyses. Allergy. (2022) 78(5):1307–18. 10.1111/all.15597 36435990 PMC10202125

[13] Saito-Abe M Yamamoto-Hanada K Pak K Iwamoto S Sato M Miyaji Y How a family history of allergic diseases influences food allergy in children: the Japan environment and children’s study. Nutrients. (2022) 14(20):4323. 10.3390/nu14204323 36297007 PMC9606927

[14] Al-Hammadi S Zoubeidi T Al-Maskari F. Predictors of childhood food allergy: significance and implications. Asian Pac J Allergy Immunol. (2011) 29(4):313–7. 22299310

[15] Venter C Warren C Samady W Nimmagadda SR Vincent E Zaslavsky J Food allergen introduction patterns in the first year of life: a US nationwide survey. Pediatr Allergy Immunol. (2022) 33(12):e13896. 10.1111/pai.13896 36564881 PMC10107094

[16] Schwarzenberg SJ Georgieff MK, Committee on Nutrition; Daniels S, Corkins M, Golden NH Advocacy for improving nutrition in the first 1000 days to support childhood development and adult health. Pediatrics. (2018) 141(2):e20173716. 10.1542/peds.2017-3716 29358479

[17] Bryant-Waugh R Markham L Kreipe RE Walsh BT. Feeding and eating disorders in childhood. Int J Eat Disord. (2010) 43(2):98–111. 10.1002/eat.20795 20063374

[18] Li XL Liu Y Liu M Yang CY Yang QZ. Early premature infant oral motor intervention improved oral feeding and prognosis by promoting neurodevelopment. Am J Perinatol. (2020) 37(06):626–32. 10.1055/s-0039-1685448 31013539

[19] Mackie RI Sghir A Gaskins HR. Developmental microbial ecology of the neonatal gastrointestinal tract. Am J Clin Nutr. (1999) 69(5):1035–45. 10.1093/ajcn/69.5.1035s 10232646

[20] McWilliam V Peters R Tang ML Dharmage S Ponsonby AL Gurrin L Patterns of tree nut sensitization and allergy in the first 6 years of life in a population-based cohort. J Allergy Clin Immunol. (2018) 143(2):644–650.e5. 10.1016/j.jaci.2018.07.038 30171872

[21] Sicherer SH Warren CM Dant C Gupta RS Nadeau KC. Food allergy from infancy through adulthood. J Allergy Clin Immunol Pract. (2020) 8(6):1854–64. 10.1016/j.jaip.2020.02.010 32499034 PMC7899184

[22] Warren C Nimmagadda SR. LEAPing into the void. Ann Allergy Asthma Immunol. (2023) 130(3):267–8. 10.1016/j.anai.2022.12.015 36868722

[23] Mikhail IJ. Implementation of early peanut introduction guidelines: it takes a village. Immunol Allergy Clin North Am. (2019) 39(4):459–67. 10.1016/j.iac.2019.07.002 31563181

[24] Trogen B Jacobs S Nowak-Wegrzyn A. Early introduction of allergenic foods and the prevention of food allergy. Nutrients. (2022) 14(13):2565. 10.3390/nu14132565 35807745 PMC9268235

